# Immunological Endotyping of Chronic Critical Illness After Severe Sepsis

**DOI:** 10.3389/fmed.2020.616694

**Published:** 2021-02-15

**Authors:** Brittany P. Fenner, D. B. Darden, Lauren S. Kelly, Jaimar Rincon, Scott C. Brakenridge, Shawn D. Larson, Frederick A. Moore, Philip A. Efron, Lyle L. Moldawer

**Affiliations:** Department of Surgery, Sepsis and Critical Illness Research Center, University of Florida College of Medicine, Gainesville, FL, United States

**Keywords:** inflammation, immunosuppression, aging, acute kidney injury, PICS

## Abstract

Improved management of severe sepsis has been one of the major health care accomplishments of the last two decades. Due to enhanced recognition and improved management of severe sepsis, in-hospital mortality has been reduced by up to 40%. With that good news, a new syndrome has unfortunately replaced in-hospital multi-organ failure and death. This syndrome of chronic critical illness (CCI) includes sepsis patients who survive the early “cytokine or genomic storm,” but fail to fully recover, and progress into a persistent state of manageable organ injury requiring prolonged intensive care. These patients are commonly discharged to long-term care facilities where sepsis recidivism is high. As many as 33% of sepsis survivors develop CCI. CCI is the result, at least in part, of a maladaptive host response to chronic pattern-recognition receptor (PRR)-mediated processes. This maladaptive response results in dysregulated myelopoiesis, chronic inflammation, T-cell atrophy, T-cell exhaustion, and the expansion of suppressor cell functions. We have defined this panoply of host responses as a persistent inflammatory, immune suppressive and protein catabolic syndrome (PICS). Why is this important? We propose that PICS in survivors of critical illness is its own common, unique immunological endotype driven by the constant release of organ injury-associated, endogenous alarmins, and microbial products from secondary infections. While this syndrome can develop as a result of a diverse set of pathologies, it represents a shared outcome with a unique underlying pathobiological mechanism. Despite being a common outcome, there are no therapeutic interventions other than supportive therapies for this common disorder. Only through an improved understanding of the immunological endotype of PICS can rational therapeutic interventions be designed.

## Sepsis Is a National Crisis

Sepsis afflicts over 1.7 million Americans annually, and accounts for over 250,000 deaths in the United States alone (1). More patients die from sepsis annually than from lung cancer, the number one cause of cancer-related deaths. Sepsis remains the most common cause of death in the intensive care unit (ICU), accounting for 1 in 3 hospital deaths (2). Sepsis is also the most expensive in-hospital diagnosis in the U.S. today (3). Even with these staggering numbers, the impact of sepsis on patients, their families, and the community is grossly underestimated as survivors experience multiple ongoing morbidities (4). Sepsis has an annual patient care cost approaching $23 billion (more than $55 million per day) (5), which again is likely an underestimate, as the chronic effects of sepsis months to years post-intensive care unit (ICU) discharge are unknown (6).

Sepsis induces a profound state of both acute and chronic immune dysregulation, which contributes to both the mortality and long-term morbidities (7). These long-lasting morbidities include frequent re-hospitalization within the year following sepsis diagnosis, with the most common admission diagnoses being pneumonia or urinary tract infection (8). Interestingly, there are no FDA-approved therapeutics for the immunologic treatment of sepsis despite over 150 clinical trials and successful pre-clinical testing (9). In-hospital management remains primarily supportive in nature. Post-hospitalization, Prescott and Angus in their 2018 *JAMA* review of enhancing recovery from sepsis had only three recommendations: (1) identify new problems and treat appropriately, (2) review and adjust long-term medications, and (3) evaluate for treatable conditions that result in rehospitalization (10). None of these approaches target the unique immunologic and physiologic consequences of critical illness, as our understanding of the acute sepsis survivor remains incomplete!

## Sepsis Is at Its Most Basic Core an Immunological Disease

Sepsis is associated with an early/immediate “systemic inflammatory response” (11). Authors have used the terms “cytokine storm” and “genomic storm” for this early response (12, 13), but these are gross over-simplifications of an integrated innate and adaptive immune response. At its most fundamental, the host response we define as “sepsis” is due to the recognition and response of host protective immune systems to microbial pathogens and their products (termed pathogen-associated molecular patterns; PAMPS) or the consequential release of endogenous alarmins (danger-associated molecular patterns; DAMPs) (14, 15). Acutely, the response appears aimed at isolating microbial growth and limiting replication, although it is well-known that when the response is either exaggerated or becomes systemic, can be associated with microcirculatory defects, organ injury, and death (16, 17).

The term “storm” is prescient, since the response, especially when the exposure is systemic, can produce devastating widespread host responses, including microcirculatory failure, profound vascular and organ injury, shock and death (18, 19). In the 1980's and early 1990's when many of the cytokines/mediators involved in this “storm” were originally identified and cloned, the complexity and breadth of the immediate host response to microbial products was not fully understood. Initial efforts to modify the host response to sepsis targeted this early/immediate response by interfering with individual microbial products and early cytokine appearance (20). Unfortunately, antibodies or immunoadhesins to endotoxin, TNFα, IL-1, IFNγ to name a very few, have all failed to improve outcomes to sepsis (21).

Although the reasons for the failure of these attempts to prevent this early “storm” are clearly multifactorial, including timing, redundancy of action, and heterogeneity of the patient population, there was also an over-assumption that treating sepsis would only require identifying the “silver bullet” responsible for the immediate organ damage (22). If there is anything that the failures of the past three decades have taught, it is that successful treatment of sepsis is, and will continue to be, an iterative process dependent upon both increasing our basic understanding of sepsis pathophysiology and translating this knowledge into improved clinical management.

What was learned was that one key to improving in-hospital survival was earlier sepsis recognition and initiation of treatment (23). Rapid diagnosis and initiation of sepsis treatment bundles have been a major hospital-systems' accomplishment (24). A second important key was the implementation of standardized best practices in the management of the sepsis patient (25). Almost two decades after implementation of the Surviving Sepsis Campaign, a 25% improvement in compliance with best-practice has resulted in a 9% absolute reduction in 28 day all-cause mortality (26). In the largest study to date, with over 1 million subjects, the state of New York-mandated early interventions significantly improved sepsis survival compared to states that did not mandate early intervention (27).

## Chronic Poor Outcomes Are Replacing In-Hospital Mortality

**Figure 1:**
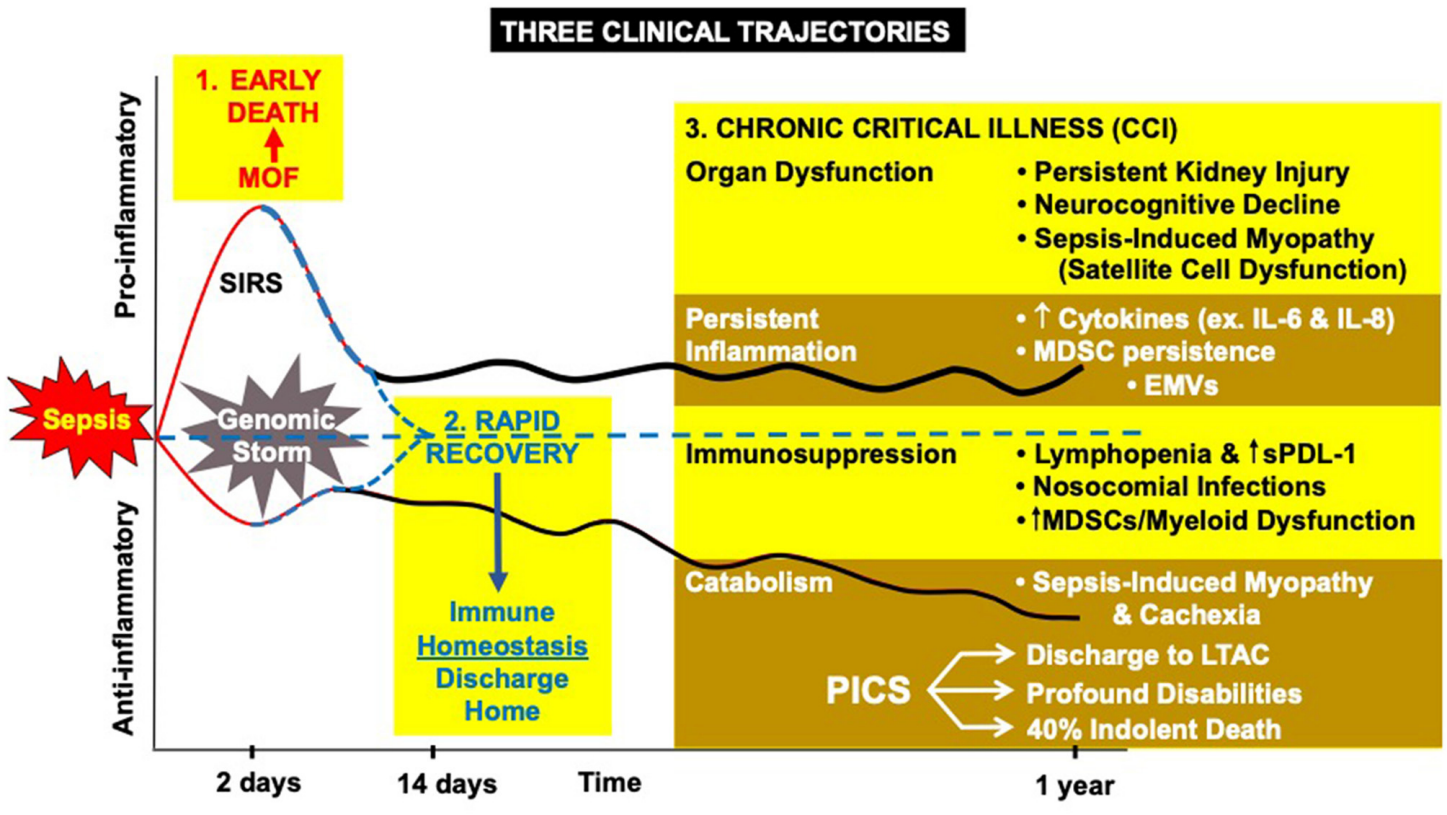
The host response to severe sepsis can have three different clinical trajectories: (1) early MOF leading to death, (2) rapid recovery, or (3) the new appearance of chronic critical illness (CCI) characterized by organ dysfunction, inflammation, immune suppression, and protein catabolism. MDSC, myeloid derived suppressor cells; EMVs, endothelial microvesicles; sPDL-1, soluble protein death ligand 1; LTAC, long-term acute care hospital.

Earlier recognition and more consistent best-practice management have resulted in fewer patients dying early from the consequences of the “storm” (28, 29). This has resulted in more in-hospital survivors, and the appearance of late immunological complications of trauma and sepsis are now becoming the norm (30). As many as 33% of all sepsis survivors do not rapidly or fully recover but, instead, develop a new syndrome of “chronic critical illness” (CCI) (31–33). Chronic critical illness is represented by a persistent low grade inflammatory and chronic immunosuppressive phase associated with functional declines that can last from months to years following the acute event (Figure 1) (32).

These mechanisms underlying post-sepsis immunosuppression and inflammation are poorly understood, limiting our ability to prevent secondary infections and improve long-term outcomes in sepsis survivors. Chronic critical illness does not have a single consensus definition, like sepsis, but all define it as ongoing/persistent manageable organ dysfunction requiring hospitalization and increased resource utilization (e.g., ICU management) (31, 34). Based upon length-of-stay based mortality data from ICU patients at Shands UF Health, we set the duration of hospitalization at 2 weeks, and this definition has gained acceptance by others (35).

Our own studies have suggested that the severity of the initial acute phase, the age of the patient, the number of pre-existing comorbidities, and the influence of kidney injury, all impact the development of CCI (36). Sepsis survivors who develop CCI experience a higher frequency of secondary infections, have longer hospital stays, and poor disposition (37). Dramatically, 60% of CCI patients are readmitted in the first 6 months (38), usually for recurrent infections, and 40% of these patients are dead at 6 months (39). Not unexpectedly, 70% of deaths are preceded by a withdrawal of care (40, 41).

## Chronic Poor Outcomes After Sepsis Are the Result of a Maladaptive Immunological Dyscrasia

Chronic critical illness represents the clinical manifestation/endpoint of a complex immunological “dyscrasia” that results in increased susceptibility to secondary infections; poor functional, physical and cognitive outcomes (34, 38, 39). We have argued that the metabolic and immunologic underpinnings of this response is the immunological endotype we have termed the Persistent Inflammatory, immunosuppressive and protein Catabolic Syndrome (PICS) (32, 42, 43). As summarized in Table 1, sepsis survivors with CCI experience the very common PICS endotype reflected by persistently elevated inflammatory cytokines and DAMPs, immune suppression, and an increased number of opportunistic infections.

**Table 1:** CCI phenotype in severe trauma and sepsis survivors.

**Phenotype**		**Sepsis CCI**
Persistent inflammation	Acute Phase Reactants—↑CRP, ↓Albumin	↑↑↑
	Cytokines	↑↑
	DAMPS, mtDNA, ncDNA, S100A8/A9, HMGB1	↑↑↑
Immune suppression	Absolute lymphocyte count	↓↓↓
	Secondary infections, sepsis readmission	↑↑↑
	MDSCs	↑↑↑
	CD14^+^ HLA-DR expression	↓↓↓
	ELISpot T-cell IFNγ	↓↓↓
Protein catabolism	Body weight loss	↑↑
	Loss of lean tissue	↑↑
	Loss of physical function	↑↑

*CRP, c-reactive protein; DAMPs, damage associated molecular patterns; mtDNA, mitochondrial DNA; s100A8/A9, calcium binding proteins that form calprotectin; HMGB1, high-mobility group box 1 protein; HLA-DR, Human Leukocyte Antigen DR isotype; IFNγ, Interferon gamma. ↑ increased, ↑↑ moderately increased, ↑↑↑ markedly increased; ↓ decreased, ↓↓ moderately decreased, ↓↓↓ markedly decreased*.

A “phenotype” is defined as a set of observable characteristics of an individual resulting from the interaction of its genotype with the environment. CCI can be classified as a phenotype because of its reproducible characteristics: ongoing manageable organ injury requiring at least 14 days of ICU care. In contrast, an endotype is defined as a subtype of a condition represented by a **distinct functional or pathobiological mechanism** (44). Importantly, endotypes differ from phenotypes because the former requires a common underlying mechanism. Endotypes, unlike phenotypes, can be associated with clusters of disease. PICS would classify as an endotype, since it may well be a common underlying mechanism that can explain not only sepsis and trauma CCI, but could also explain in part, cancer cachexia, and the chronic inflammation and lean tissue wasting associated with chronic obstructive pulmonary disease, cardiac cachexia and chronic renal disease.

The immunological dyscrasia that defines PICS is multifactorial. Primary mechanisms leading to sepsis-induced impairment of adaptive immune system include: (i) apoptosis-induced T-cell depletion, (ii) T-cell exhaustion due to upregulation of inhibitory receptors or downregulation of essential co-stimulatory receptors, (iii) decreased bone marrow lymphopoiesis, and (iv) myeloid-based T-cell suppression, and myeloid cell dysregulation (45–47).

“Emergency myelopoiesis” is defined as inflammation-induced hematopoiesis, which is critical for the immediate management of tissue injury and controlling infection (48–50). In contrast to adaptive immune cells, such as T cells and B cells, that proliferate in response to their specific antigens, innate myeloid populations are continuously replenished from hematopoietic stem cells (HSCs) and progenitors in bone marrow (BM) and extramedullary (42, 51). However, the molecular mechanism of emergency myelopoiesis during infection remains incompletely understood. HSCs and hematopoietic progenitors can directly sense the presence of pathogens or endogenous alarmins *via* pattern recognition receptors (PRRs) such as Toll-like or NOD-like receptors (TLRs, NLRs), and they can also respond to pro-inflammatory cytokines such as interferon (IFN)-α, IFN-γ, interleukin (IL)-1, tumor necrosis factor (TNF)-α, and granulocyte colony-stimulating factor (G-CSF) (42). IFN-α and IFN-γ have pleiotropic effects on many cell types, including HSCs and hematopoietic progenitors (52). Importantly, these cytokines, along with IL-27, have been demonstrated to induce an expansion of HSCs and myeloid progenitors, leading to the production of differentiated PMNs, macrophages and dendritic cells at the cost of both lymphopoiesis and erythropoiesis (49, 50).

**Figure 2:**
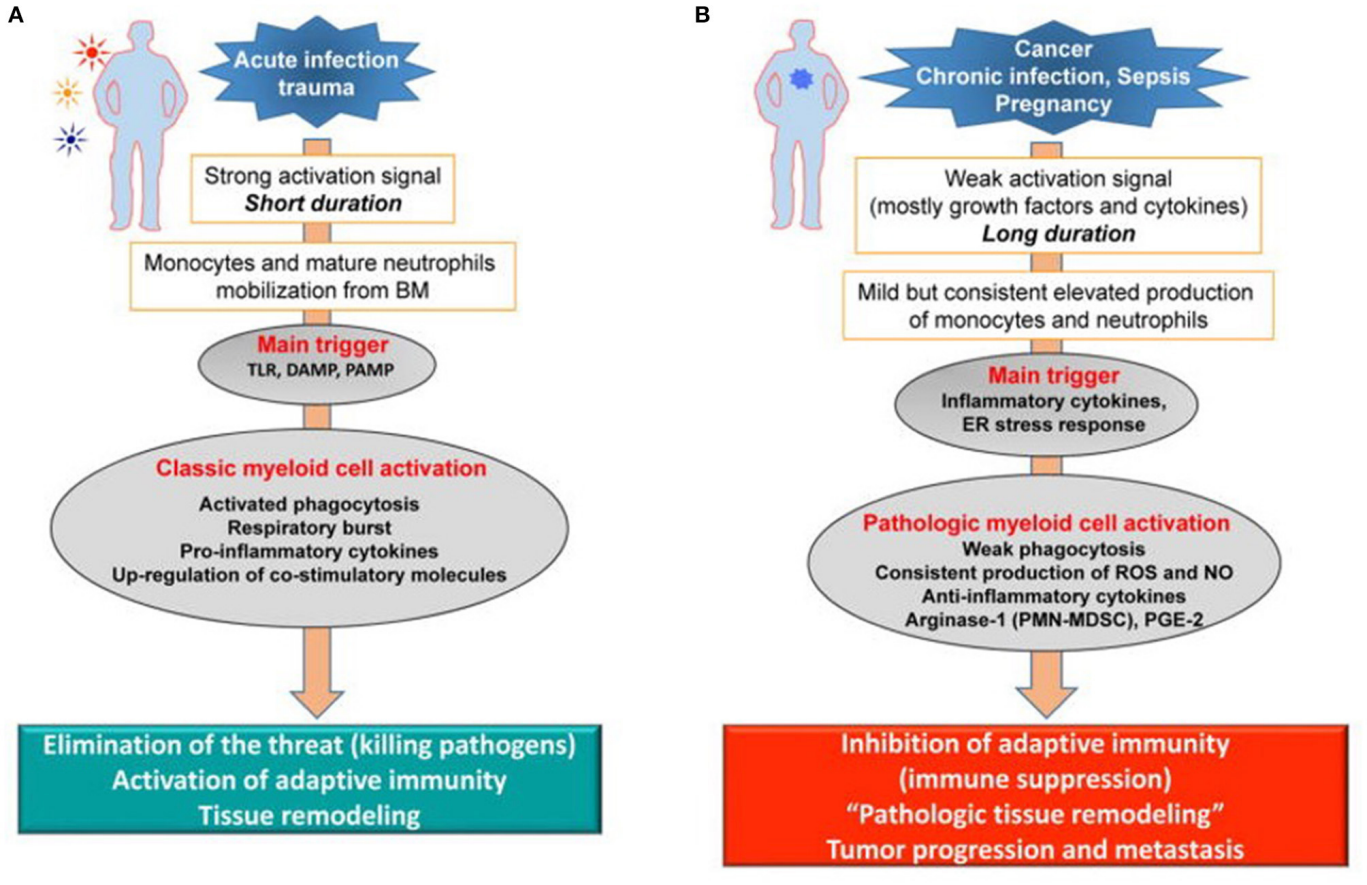
Pathologic activation of neutrophils and monocytes. **(A)** In the presence of strong activation signals coming from pathogens in the form of toll-like receptors ligands (TLRL), damage associated molecular pattern (DAMP), pathogen-associated molecular patterns (PAMP) molecules monocytes and neutrophils are mobilized from the BM. This response results in classic myeloid cell activation. **(B)** In the presence of weak activation signal mediated mostly by growth factors and cytokines, myeloid cells undergo modest but continuous expansion. Pro-inflammatory cytokines and ER stress responses contribute to pathologic myeloid cells activation that manifests in weak phagocytic activity, increased production of reactive oxygen species (ROS), nitric oxide (NO), arginase 1 (not expressed in human monocytes and M-MDSC) and prostaglandin-E2 (PGE2). This results in immune suppression. Endoplasmic reticulum = ER. Modified from Veglia et al. (53).

Dmitry Gabrilovich has argued that the long-term host response to cancer, chronic infection, and sepsis (Figure 2B) results in what he has termed “pathological activation of neutrophils and monocytes” (53). Weak activation signals that occur in sepsis survivors during CCI, such as endogenous alarmins released from low grade organ injury, secondary colonization and infection from invasive ICU procedures, immobility, and delirium, all result in the mild but consistent elevated production of inflammatory cytokines and signals that drive persistent “emergency myelopoiesis.” What Gabrilovich has argued (54), and we have experimentally demonstrated in both trauma and sepsis (55), is that long-duration, low-grade inflammation drives pathologic myeloid cell activation and T-cell exhaustion, leading to both persistent inflammation and immune suppression (PICS).

During unresolved inflammation, such as following microbial infection, ongoing tissue injury, and other chronic conditions; the nature of signals affecting T-cells and myeloid cells differs from that seen during the early or immediate “genomic or cytokine storm” (56). Reduced bone marrow and thymic generation of new T-cells and increased expression of immunosuppressive receptors favors exhaustion and apoptosis of T-cell populations resulting in lymphopoiesis. The expansion of myeloid-derived suppressor cells (MDSCs) in sepsis is a complex and gradual phenomenon governed by multiple factors. Gabrilovich has argued that accumulation of MDSC requires two groups of signals: the first leading to expansion of immature myeloid cells and the second, pathological activation as MDSCs. The first group of signals is driven as a direct host response to the microbial pathogen and includes: GM-CSF, G-CSF, M-CSF, SCF, VEGF, and polyunsaturated fatty acids (PUFAs) (53, 57). Transcriptional factors/regulators including STAT3, STAT5, IRF8, C/EBPβ, and NOTCH play a major role in this process (58). Other factors involved in this process include adenosine receptors A2b, NLRP3, and alarmins S100A8 and A9 (59). Importantly, the second group of signals, resulting in the pathological activation of MDSCs, does not require an infectious process and can be provided by inflammatory cytokines and endogenous alarmins alone, which include interferon (IFN)-γ, IL-1β, IL-4, IL-6, IL-13, IL-27, TNF-α, and the TLR ligand, HMGB1 (59).

First and foremost, these stimuli drive the expansion of bone marrow and extramedullary myelopoiesis. Neutrophils and monocytes generated under these conditions display a variant phenotype and morphology. They are characterized by relatively weak phagocytic activity, increased levels of reactive oxygen species (ROS) and nitric oxide (NO) production, and high expression of arginase 1, PGE_2_, and a number of anti-inflammatory cytokines (60, 61). Most of these features are absent in classically activated neutrophils and monocytes, which is why Gabrilovich has characterized this activation as “pathologic” (54). This state of activation leads not to the elimination of the threat or activation of host protective immunity, but to the inhibition of adaptive and innate immunity. Cells in this pathologic state of activation can be identified functionally, biochemically, and, to some extent, phenotypically, and are now termed MDSCs. The longer the myeloid compartment is exposed to the effects of factors described above, the more potent the pathologic activation of these MDSCs. Therefore, at any given moment, there is a heterogeneous population of cells in tissues represented by classically activated neutrophils, monocytes, and pathologically activated MDSCs (Figure 2). In the early stages of sepsis, bona-fide immune suppressive MDSCs are rarely detected (62, 63). However, there are cells with some biochemical and genomic characteristics of MDSCs, which probably represent an intrinsic part of MDSC development (53, 62).

The evidence that expansion of immunosuppressive MDSCs is a constant response to prolonged sepsis is incontrovertible. As early as 2007, we demonstrated that by 7 days post-sepsis, up to 95% of bone marrow cells are of myeloid lineage, mostly immature and functional MDSCs (64). These cells also overwhelm secondary lymph tissues, such as the spleen, lymph nodes, and reticuloendothelial tissues (such as the lung and liver) (65). Importantly, we have demonstrated that rapid, sustained presence of MDSCs, and their quantitative levels are strong predictors of nosocomial infections and poor discharge outcomes in sepsis patients (7). These findings were confirmed by Uhel, who established that sepsis survivors with expanded MDSC populations had a higher rate of reinfection and hospital readmission (66).

**Figure 3:**
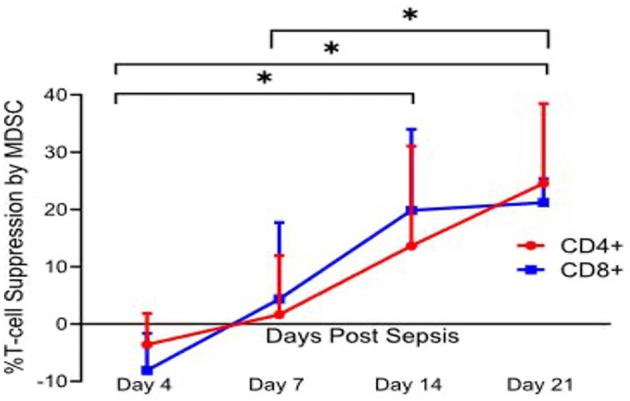
Percent T lymphocyte suppression by MDSCs. Immature myeloid cells with the surface markers CD33^+^CD11b^+^HLA-DR^dim^ were isolated on days 4, 7, 14, and 21 after sepsis, and from healthy control subjects. Autologous T lymphocytes were stimulated with soluble anti-CD3/28 and seeded in a co-culture with MDSCs in a 1:1 ratio. T cells were labeled with CellTrace Violet to detect proliferation, and a proliferation index (PI) was calculated for both CD4^+^ and CD8^+^ T cells using flow cytometric analysis. Percent suppression was calculated as the ratio of PI from stimulated T cells in the presence of MDSCs and the PI of stimulated T cells in culture medium alone. Percent suppression for both CD4^+^ and CD8^+^ T cells was significantly different between day 4 vs. 14 (*p* = 0.0402 and 0.0012), day 4 vs. 21 (*p* = 0.0225 and <0.0001), and day 7 vs. 21 (*p* = 0.037 and 0.045). There was no significance noted of percent suppression of CD4^+^ and CD8^+^ T cells between days 7 and 14 (*p* = 0.17 and 0.08). This T cell suppression was not seen in age-matched healthy control subjects. Modified from Hollen et al. (62). *indicates statistically significant intervals (*p* < 0.05).

Additionally, McCall et al. demonstrated that these cells evolve functionally over time, becoming more immunosuppressive (32). With regard to MDSC suppressor activity, Hollen et al. have observed considerable time-dependent differences in MDSC suppression of T-cell proliferation. Much to our surprise, but consistent with Gabrilovich's overarching hypothesis (Figure 2), pathological activation of MDSCs in humans did not occur immediately after sepsis, but required 7–14 days to develop fully. Regardless of whether MDSCs came from sepsis survivors who developed CCI or rapidly recovered, PBMC-derived CD11b^+^CD33^+^HLA-DR^dim^ MDSCs obtained prior to day 7 were not immunosuppressive, while MDSCs obtained at or after day 14 (all CCI patients) suppressed both autologous CD4^+^ and CD8^+^ T-cell proliferation to antiCD3/CD28 (Figure 3). Also, septic MDSCs from day 14 (late), but not from day 4 (early), potently suppressed stimulated T-cell production of IL-2 and, to a lesser extent, IFNγ.

**Figure 4:**
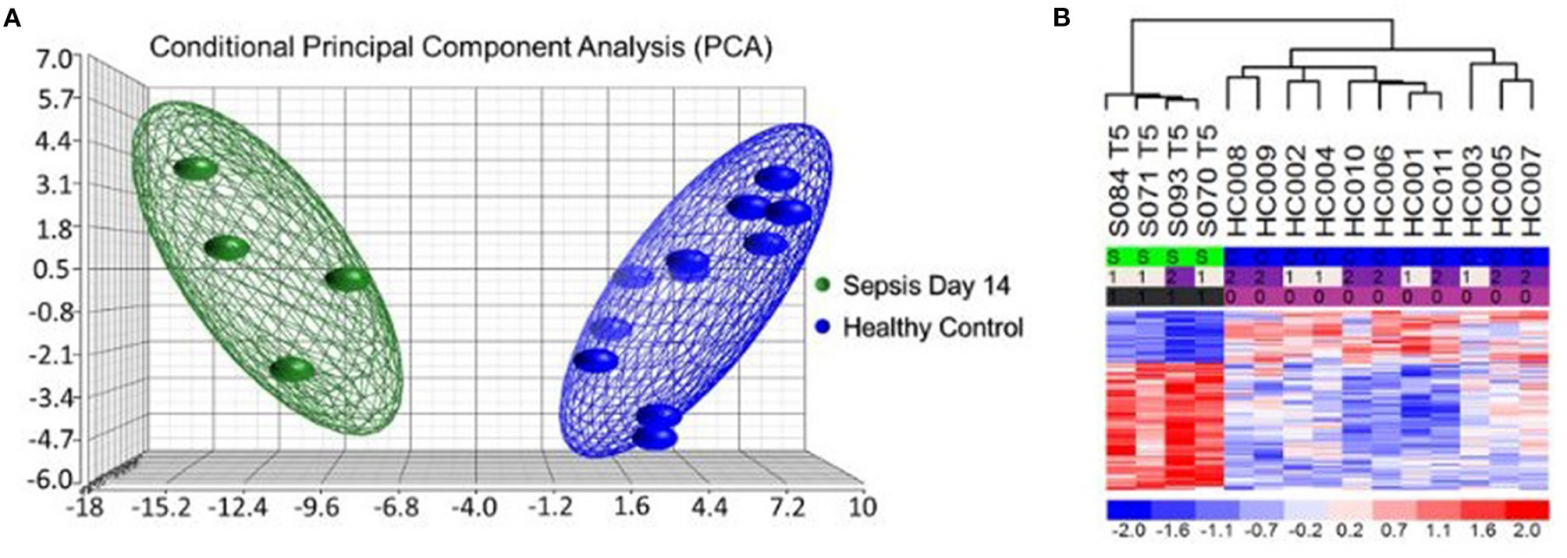
Microarray Transcriptomic Analysis of MDSCs from Patients 14 days after Sepsis and Healthy Control Subjects. The genomic response of bulk isolated MDSC RNA in healthy controls and septic patients 14 days after initial infectious onset. **(A)** Conditional principal component analysis of septic (day 14) and healthy control MDSC gene expression patterns. **(B)** Heat map of the hierarchical clustering of MDSC gene expression patterns and variation between septic patients (S) from day 14 and healthy (H) control subjects. Modified from Mathias et al. (67).

More interestingly, most of the MDSCs in septic CCI patients were granulocytic with a gene expression profile reflective of a highly inflammatory and immunosuppressive transcriptome (67). Analysis of individual gene transcripts from bulk cell-sorted human CD11b^+^CD33^+^HLA-DR^dim^ MDSCs was consistent with suppressed HLA gene expression and up-regulated inflammatory gene expression (Figure 4). Canonical Pathway and Causal Network Analysis supported these pathway alterations and a pattern of simultaneous low-grade inflammation with immunosuppression.

**Figure 5:**
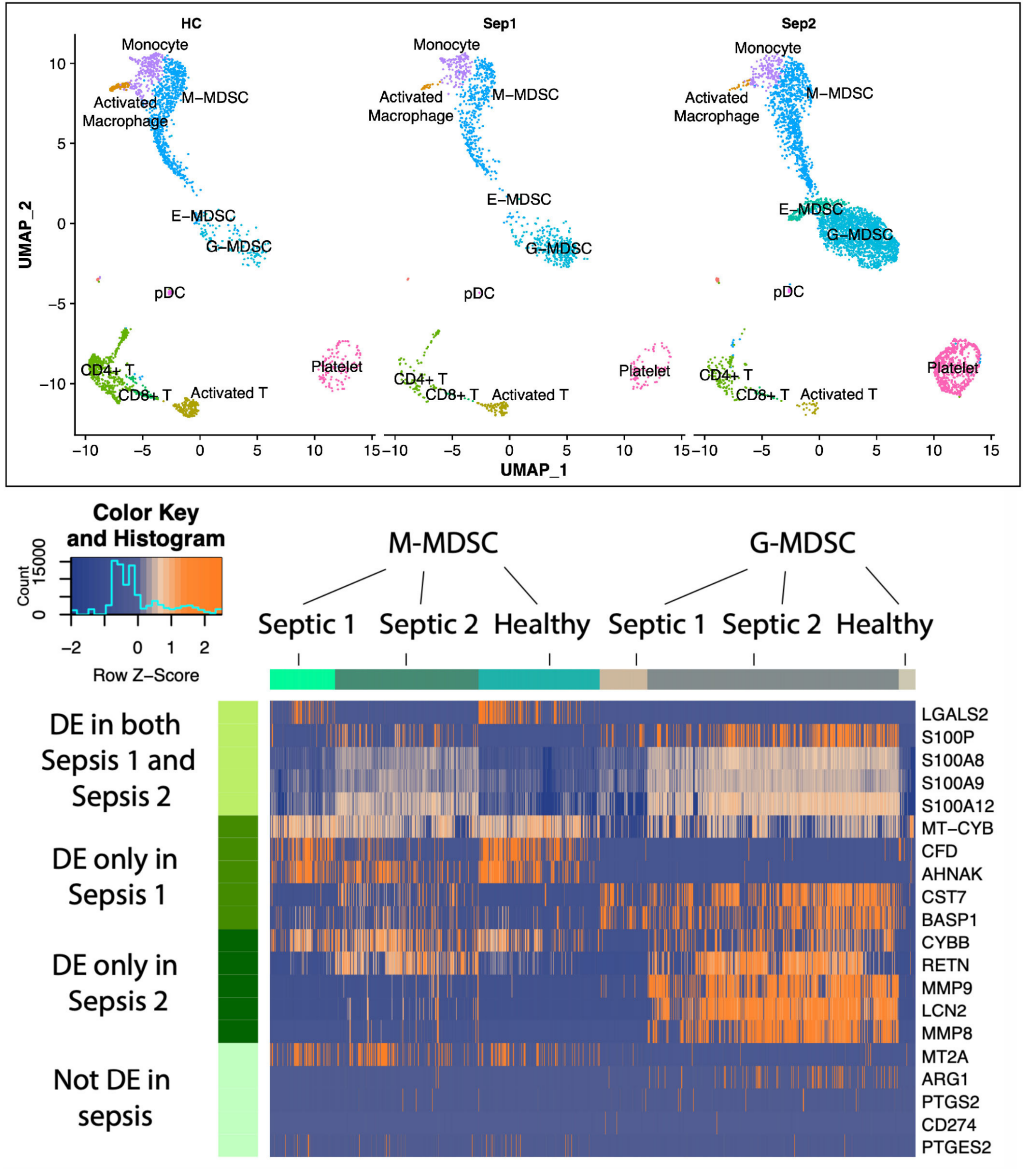
Uniform manifold approximation and projection (UMAP) plots of cell clusters identified in healthy patients (*n* = 2,340 cells) vs. sepsis 1 (bacteremia, sepsis; *n* = 1,544 cells) and sepsis 2 (fungemia, septic shock; *n* = 5,587 cells) showing three distinct MDSC subsets. Heatmap illustrating expression patterns of MDSC subsets at 21 days post-sepsis vs. healthy control subjects. Rows represent the specific genes of interest differentially expressed in both sepsis patients; only sepsis patient 1; only sepsis patient 2; and genes not differentially expressed in this study, but previously determined to be important to MDSC function in cancer and autoimmunity. Number of columns represent number of cells analyzed in each group. DE, differentially expressed genes in sepsis vs. healthy controls. Colors represent mean normalized relative expression with blue representing reduced expression and orange, increased expression. Modified from Darden et al. (68).

Although G-MDSCs (granulocyte-like MDSCs) comprise the largest subpopulation of MDSCs in sepsis, expansion of M-MDSCs (monocytic MDSCs) and E-MDSCs (early MDSCs) is also observed. Importantly, different subpopulations of MDSCs are immunosuppressive through different mechanisms, and can, therefore, have different targets for intervention (53). To understand the rich “landscape” of blood MDSCs late after sepsis (day 21), single-cell RNA sequencing and Cellular Indexing of Transcriptomes and Epitopes by Sequencing (scRNA-seq and CITE-seq) was conducted on enriched MDSCs obtained from peripheral blood mononuclear cells (PBMCs) (Figure 5). This was conducted to identify individual populations of MDSCs (G-, M-, and E-MDSCs) and their transcriptomic profiles in healthy and septic patients. In this case, samples were obtained at day 21 from two sepsis survivors with CCI, and samples were also obtained from two age and sex matched healthy, control subjects. Samples were first isolated on a Ficoll gradient, and then CD11b^+^CD33^+^HLA-DR^dim^ cells were mixed 3:1 with original PBMCs to assure inclusion with all cell populations. sc-RNAseq of over 150,000 cells were conducted.

As displayed in Figure 5, there was a dramatic expansion of the G-MDSC subpopulation and a less dramatic expansion of M-MDSCs in the sepsis patients. E-MDSCs, which were not detectable from healthy human subjects, were modest in sepsis survivors with CCI (detectable in only one of the sepsis subjects). G-MDSCs showed not only the greatest expansion, but also the most dramatic changes in their transcriptome. Interestingly, we did not see an increase in expression among genes that are associated with immunosuppression in cancer (such as *ARG1, CD274, COX2, PGE2*, and *NOS2*). While this was a small pilot study, it suggests that MDSCs present in sepsis may be inherently different from those seen in cancer-associated immunosuppression.

## Aging Predisposes the Development of CCI and PICS

The global population is rapidly aging (70, 71) with increasing healthcare resources and costs devoted to this group. The frequency of hospitalizations for sepsis in patients over 50 has increased, most dramatically in patients aged 65 years or greater (72). Advanced age is also associated with more severe organ failure, infectious complications, increased ventilator days, a longer ICU LOS, an increased 28-day mortality, and an increased likelihood of discharge to skilled nursing or long-term care facilities (73).

As seen in younger populations, the in-hospital mortality from sepsis in the elderly is decreasing, but still remains significantly higher. The risk of CCI and discharge to a non-home destination is also increased in the elderly population and is multifactorial in nature (33, 72, 74). Contributing factors include senescence (normal aging), inflammaging (chronic, subclinical inflammation), comorbidities, lack of physiologic reserve, pre-existing disability, and epigenetic changes. These factors prevent older individuals from readily returning to homeostasis following critical illness and contribute to the increased risk of morbidity and mortality following sepsis (70, 71, 75, 76).

Aging has a profound role on the immune system. Immunosenescence is a state of age-associated changes in the immune system which is characterized by decreased ability to mount an effective response to pathogens (77, 78), decreased competency of the adaptive immune system (as evidenced by decreases in naïve peripheral T cells, repertoire diversity, and immunocompetent B cells) (77, 79), and dysfunctional myelopoietic effector cells (i.e., PMNs, monocytes/Mϕ, DCs, and NK cells) (78). Furthermore, the aged host's HSCs preferentially induce myelopoiesis, contributing to the substantial increase in MDSCs seen in this population as a response to the initial insult (78). “Inflammaging” is defined as chronic, low-grade inflammation that occurs with physiologic aging. It is a unique response seen in aged mammals and differs from the responses seen in the young. The “cytokine storm” seen in the younger population can be markedly attenuated or absent in the aged population, whereas immunosuppression appears to dominate (73). In these cases, early mortality is due, instead, to failure of host protective immune mechanisms to adequate microbial control.

In murine models, sepsis induces a rapid release of mature and immature myeloid cell populations from the bone marrow in response to endogenous and exogenous danger signals (55, 60). This creates niches in the bone marrow, which stimulate emergency myelopoiesis (80). Myelopoiesis predominates at the expense of lymphopoiesis and erythropoiesis (64, 80). Interestingly, elderly HSCs have this phenotype and function prior to critical illness, with myeloid-skewed cell production and a decreased ability to produce lymphoid cells (81, 82). These HSCs are also functionally inferior to their younger counterparts, with a lower functional frequency, delayed proliferative response, and reduced efficiency for short term homing (81, 82). These baseline dysfunctions are exacerbated by acute critical illness.

## Muscle Wasting and Protein Catabolism Sustain The CCI Response

**Figure 6:**
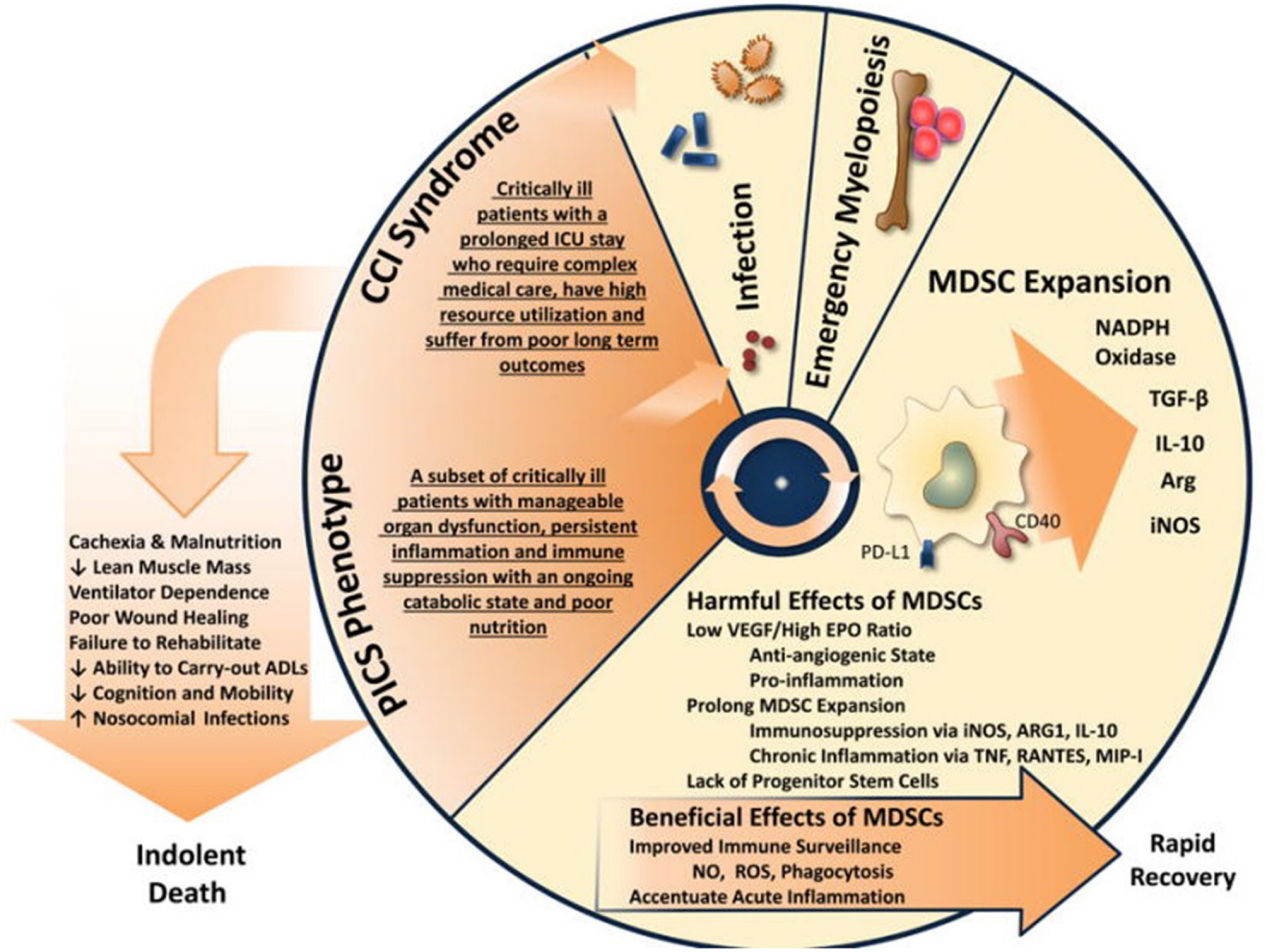
The proposed self-perpetuating cycle by which PICS drives muscle loss and inflammation. Modified from Mira et al. (69).

Skeletal muscle serves as the largest protein reserve in the body, which can be mobilized for metabolic substrates in times of stress. Critical illness is characterized by marked protein catabolism, which results from increased muscle breakdown, decreased protein synthesis, and the release of potential pro-inflammatory degradation products (83, 84). In patients who progress to CCI, this is a self-perpetuating cycle that results in profound cachexia. The exact mechanism has not been fully elucidated, but likely involves inflammation- and oxidation-associated direct mitochondrial and myocyte injury (85). Not surprisingly, muscle catabolism can result in the release of DAMPs (including mtDNA, HMGB1, and TFAM) into the systemic circulation, driving persistent inflammation (83, 86–88). In animal models, the mtDNA-TLR9-RAGE pathway, which can be activated by mtDNA or TFAM, has been shown to be involved in sepsis-induced cardiac inflammation (86, 89). As with other endogenous alarmins, these increases in both local tissue damage and systemic inflammation drives ongoing functional immunosuppression at both the level of the bone marrow (enhanced myelopoiesis) and functional lymphocyte populations (Figure 6). The role of MDSCs in cancer cachexia has been investigated for years, and several therapies are targeted at altering their function and have shown promise (90, 91).

Muscle wasting not only contributes to the ongoing inflammatory state seen in CCI, but also leads to a substantial functional disability (92, 93). Loss of skeletal muscle mass is associated with profound functional deficits which are most notable in the aging due to their baseline declining muscle mass (94). In these populations, even small functional changes can cause a shift from independent to dependent living (95). Decreased skeletal muscle mass is also associated with increased falls in the elderly, which are an independent predictor of 1-year mortality (96). Functional declines aren't limited to the aging population—over 80% of critical illness survivors report a reduction in physical ability (97). Physical limitations preclude patients from returning to work and contribute substantially to their reduced healthcare-related quality of life (97, 98). As seen with the self-perpetuating cycle of PICS in CCI patients, reductions in physical capabilities propagate ongoing mental health issues, economic hardship, and burden on the healthcare system.

## Role of Acute Kidney Injury

Organ injury contributes to the ongoing inflammation associated with CCI. The kidney plays a crucial role in both initial and long-term survival from sepsis. AKI has been associated with increased in-hospital mortality and is more prevalent in sepsis than other critical illnesses (99). This relationship is bidirectional, as patients with acute and/or chronic kidney injury are also more likely to develop sepsis (100–103). Failure to resolve AKI is associated with both increased risk of initial mortality and progression to CCI (33, 104, 105). Chronic kidney disease (CKD) is not just a marker of CCI but perpetuates the cycle as well. Renal tubule epithelial cells are highly susceptible to oxidative stress and release large quantities of DAMPs. Urinary analysis of septic patients reveals increased levels of DAMPs and over expression of several DAMP receptors (106). These DAMPs act locally to increase secretion of chemokines by renal parenchymal and dendritic cells (DCs), which further promote local inflammation (107–109). They also have systemic effects, which are mediated by PPR toll-like receptors (TLRs) (110). TLRs are also upregulated in AKI *via* epigenetic remodeling, priming the renal tubule epithelial cells to release increased amounts of cytokines in response to antigen stimulation (111). This “hyper-responsive state,” concomitant with decreased renal clearance, leads to amplified systemic inflammation and resultant organ injury (112, 113).

While the relationship between AKI and acute illness is well-established, the role of the kidney in CCI is more elusive. Its role in filtration exposes the kidney to over 30 times the blood volume daily (114), meaning renal DCs and lymph nodes are exposed to inflammatory mediators and pathogens significantly more than other tissues. This results in a positive feedback loop in which exposure to these stimuli result in further oxidative damage and release of additional inflammatory mediators, both as a response to the filtered pathogens and to the ongoing tubular necrosis (115). As previously discussed with sepsis-associated muscle wasting, these self-perpetuating cycles of ongoing cell death and inflammation result in development of a persistent inflammatory state rather than return to homeostasis.

## Investigative Challenges

Sepsis is, at its foundation, a heterogenous disease process that occurs in a diverse patient population, especially aged and those with pre-existing comorbidities. The resulting CCI in many sepsis patients is inherently intertwined with the patient's prior comorbidities and functional status. This complex pattern of seemingly infinite variables makes both clinical decision-making and systematic investigation difficult. Human studies are difficult to standardize and depend on long-term participation of patients, many of whom are overburdened with their disease process.

Animal models have been generally successful in the investigation of early sepsis responses associated with the “genomic or cytokine storm.” However, they fall short in modeling long-term processes. To better investigate these chronic mechanisms driving CCI, continued bidirectional translational research is required. Our group, along with several others, have proposed a semi-lethal cecal ligation and puncture (CLP) model with daily chronic stress to approximate the PICS endotype seen in human CCI (116–121). However, this model fails to accurately represent the sterile inflammation present in most CCI patients, and replaces it with a peritoneal abscess (122). This model also relies on young, otherwise healthy mice. It fails to capture the interplay between aging and comorbidities demonstrated in human patients. We have demonstrated that the sepsis response is notably different in aged mice when compared to their juvenile counterparts (123). Understanding these complex, interconnected mechanisms is crucial to further understanding of this disease process and development of therapeutic interventions.

## Moving Forward

Sepsis and CCI are immune dyscrasias at their foundation, and PICS is the predominant endotype behind CCI. Thus, a large majority of interventional studies have focused on the restoration of immune system homeostasis. Leukocyte growth factors (e.g., G-CSF) (124–128) immunomodulatory cytokines (e.g., IL-7, IL-15, and IFN-γ) (129–133) inhibitors of negative co-stimulatory pathways (e.g., anti-PD-1/PD-L1 Ab, anti-CTLA-4 Ab, anti-TIM3 Ab, and anti-LAG-3 Ab) (134–140) and the thymic peptide thymosin-α1 (141) have all been or are being investigated for potential benefits. These trials have been largely unsuccessful at finding a “silver bullet” cure, but some have shown promise in selective populations (124, 125). This is not surprising given the heterogenous disease process and highlights the importance of continued investigative efforts in endotyping these patients as a precision medicine approach. Oncology research has been successful in developing targeted therapies for specific cancer patients with PICS-like endotypes. These approaches may be applicable to sepsis-induced PICS, but further research is required.

Nutritional support is paramount in both the acute and chronic treatment of sepsis and CCI. As discussed before, the loss of skeletal muscle contributes to both the functional declines seen in CCI patients and the perpetuation of the PICS endotype. Early implementation of nutrition is clearly important, but the optimal protein and nutrient requirements remain undetermined. However, given their phenotypic similarities to cancer cachexia and aging sarcopenia, CCI patients likely have a daily protein requirement of roughly 1.5–2.0 g/kg/day (142–146). Arginine supplementation in sepsis remains controversial given its role as an intracellular substrate for nitric oxide. However, the upregulation of arginase-1 by MDSCs may result in a relative arginine deficiency (67, 147). Arginine is necessary for proper T-cell receptor function and wound healing (148, 149). Therefore, arginine supplementation may counteract the persistent arginine deficiency due to persistent MDSC expansion during PICS, promoting lymphocyte proliferation and improved tissue repair. Leucine is another amino acid that shows promising results, as it decreases muscle protein catabolism and induces protein synthesis (150). Leucine and other branched chain amino acids (BCAA) supplementation resulted in improved nutritional and immunologic parameters, such as nitrogen balance, prealbumin levels, and lymphocyte counts (151). It has also been shown to increase muscle protein synthesis through the mTOR pathway (152, 153). Studies in large burns have also shown promise using adjuncts such as insulin, oxandrolone, and propranolol to maintain an anabolic state (154–156).

Decreases in functional status are closely associated with decreases in health-related quality of life (QOL) among CCI patients. Maintaining, or improving, baseline functional status is the ultimate goal, but the prevention of unnecessary muscle loss is vital. Early ICU-based exercise and physical therapy programs have been associated with improved in-hospital outcomes, such as a reduction in the duration of mechanical ventilation and ICU length of stay. They are also associated with improved physical function after discharge (157). These programs, when combined with adequate nutritional support have demonstrated substantial improvements in muscle synthesis and functional outcomes (158). However, these have not been fully evaluated in the CCI population.

Technology and the field of medicine have developed rapidly over the past few decades. With the application of the Human Genome Project and the development of high throughput sequencing techniques, the development of individualized therapies has been made possible. These therapies have been remarkably successful in the treatment of cancer and congenital disease (159–161). Understanding the transcriptomic landscape of sepsis and CCI, and how they differ, is crucial to development of novel therapeutic agents for sepsis-induced CCI. Advances in technology have also made data collection and interpretation easier, making large, multicenter databases commonplace. The addition of biologic variables, in addition to clinical variables, will likely improve the prognostic power of these data sets and allow for early endotyping of patients (162–164).

“Big Data” is particularly useful in sepsis and CCI, as the disease process and patient population are increasingly heterogeneous. With the ability to quickly endotype a patient, accurate prognosis and optimal treatment is possible. We have shown recently that the leukocyte transcriptome within 48 h post-trauma is highly predictive of outcomes (165, 166). This technique, using regression-based prediction models, may be further improved by the use of machine-learning algorithms and deep-learning technologies (167, 168). Big data provides large sample sizes allowing for the identification of biomarker cutoff values with optimal sensitivity and specificity. However, these static thresholds fail to account for individual physiology; therefore, it is important that future efforts continue to improve upon precision medicine by integrating data from multicenter and multinational repositories with machine-learning and deep-learning technologies.

## Author Contributions

BF, DD, LK, PE, and LM drafted the manuscript. JR, SB, SL, and FM provided critical revisions. All authors made substantial contributions to the conception and design of the work, approved the submitted version of the manuscript, and agree to be accountable for all aspects of the work.

## Conflict of Interest

The authors declare that the research was conducted in the absence of any commercial or financial relationships that could be construed as a potential conflict of interest.
